# Supplementary material for: miRNA family miR-29 inhibits PINK1-PRKN dependent mitophagy via ATG9A
Source: bioRxiv. 2024 Jan 19:2024.01.17.576122. Preprint. [Version 1] doi: 10.1101/2024.01.17.576122 (PMC10827147; doi:10.1101/2024.01.17.576122)
Supplement: Supplement 1 [file media-1.pdf]

SUPPLEMENTARY INFORMATION

miRNA family miR-29 inhibits PINK1-PRKN dependent mitophagy via ATG9A

Briana N Markham[1], Chloe Ramnarine[1], Songeun Kim[1], William E. Grever[2], Alexandra I Soto-Beasley[1], Michael Heckman[3], Yingxue Ren[4], Andrew C Osborne[5], Aditya V Bhagwate[5], Yuanhang Liu[5], Chen Wang[5], Jungsu Kim[1, #], Zbigniew K Wszolek[6], Owen A Ross[1,7], Wolfdieter Springer[1,7,$], and Fabienne C Fiesel[1,7,$,*]

[1] Department of Neuroscience, Mayo Clinic, Jacksonville, FL 32224, USA
[2] Revvity, Waltham, MA 02451, USA
[3] Division of Clinical Trials and Biostatistics, Mayo Clinic, Jacksonville, FL 32224, USA
[4] Department of Quantitative Health Science, Mayo Clinic, Jacksonville, FL 32224, USA
[5] Department of Quantitative Health Science, Mayo Clinic, Rochester, MN 55905, USA
[6] Department of Neurology, Mayo Clinic, Jacksonville, FL 32224, USA
[7] Neuroscience PhD Program, Mayo Clinic Graduate School of Biomedical Sciences, Jacksonville, FL 32224, USA
[#] present address: Department of Medical and Molecular Genetics, Stark Neurosciences Research Institute, Indiana University School of Medicine, Indianapolis, IN 46202, USA

[$] these authors contributed equally
* Correspondence: Fiesel.Fabienne@mayo.edu

FIGURE LEGENDS & TABLES

Figure S1. High content imaging assay development.

(**A**) GFP-PRKN cells were transfected with a non-targeting siRNA, a negative control miRNA, or transfection reagent only and assessed for the percentage of GFP-PRKN translocation positive cells at increasing CCCP concentrations. Shown is the mean ± SEM of duplicate wells from one independent experiment. (**B**) Representative images show classification of cell populations into GFP-PRKN translocation positive (red) versus negative cells (green). Border elements were excluded from the analysis.

Figure S2. Validation of the RNAseq results.

(**A**) Four genes with varying fold changes were chosen for general validation of RNA sequencing. (**B**) Cells were transfected with miR-29a and analyzed for fold change of mRNA for the chosen genes of COL4A1, HMGCR, CDC42, and VEGFA. Shown is the average fold change for three sets of experiments normalized to the control miRNA + SEM.

Figure S3. Effects of miR-29 on pS65-Ub and PINK1.

(**A**) HeLa cells transfected with miR-29a, miR-29b or miR-29c were analyzed using western blot for pS65-Ub signal after 2 h CCCP treatment. Shown is the mean normalized to GAPDH + SEM from at least four independent experiments. Statistical analysis was performed using two-way ANOVA with Dunnett's multiple comparisons test. (**B**) HeLa cells transfected with miR-29a, miR-29b, and miR-29c and analyzed for fold change of mRNA levels for PINK1 via qRT-PCR. Shown is the average fold change normalized to the control + SEM from at least four independent experiments. Statistical analysis was performed using one-way ANOVA with Dunnett's multiple comparisons test. (**C**) PINK1 levels were also analyzed using immunoblot analysis. Shown is the

mean normalized to GAPDH + SEM. Statistical analysis was performed using two-way ANOVA with Dunnett's multiple comparisons test.

Figure S4. Silencing of ATG9A reduces mitophagy.

(**A**) Live cell high content mitolysosome analysis of HeLa GFP-PRKN MitoKeima cells transfected with scrambled siRNA control, ATG9A siRNA, or PINK1 siRNA treated with CCCP before imaging. Total area of acidic mitochondria over total mitochondria per cell was analyzed every two hours for the first six hours and then every four hours for a total of 18 hours. Shown is the mean of 18 replicate wells from one experiment ± SEM. Statistical analysis was performed using two-way ANOVA with Dunnett's multiple comparisons test. (**B**) Shown are representative 20x zoomed images of mitolysosomes for each of the timepoints for scrambled siRNA control (scr), ATG9A siRNA, and PINK1 siRNA transfected wells. Acidic mitochondria are colored red and neutral mitochondria are colored green. Scale bars represent 20 µm.

Figure S5. ATG9A and miR-29a both increase lipid droplet number and size.

(**A**) HeLa cells were transfected with either scrambled control siRNA or ATG9A siRNA and stained with the nuclear stain Hoechst and the lipid droplet probe BODIPY 493/503. Shown are representative images after treatment with 0, 2, and 4 h CCCP. (**B**) Shown is the mean ± SEM from three independent experiments of lipid droplet puncta number per cell and the mean lipid droplet area per cell. (**C**) HeLa cells were transfected with negative control miRNA or miR-29a and stained with Hoechst (blue) and the lipid droplet probe BODIPY 493/503 (green). Shown are representative images after treatment with 0, 2, and 4 h CCCP. (**D**) Shown is the mean + SEM from three independent experiments of lipid droplet puncta number per cell and the mean lipid droplet area per cell. Statistical analyses were performed using an unpaired t-test. (**E**) HeLa cells

were stained with ATG9A, mitochondrial marker HSP60, and nuclear marker Hoechst. Shown are representative images after 0, 2, and 6 h CCCP treatment. Scale bars represent 10 µm.

Table S1. Imaging properties considered to find the best descriptor for PRKN translocation.

Table S2. Image analysis routine for miRNA screen.

Table S3. Final linear classifier to define PRKN translocation positive cells.

Table S4. Conserved 3'-UTR miR-29 binding sites and alignment of ATG9A to miR-29a/b/c.

Table S5. ATG9A species alignment at the R631 and S828 positions.

Figure S1

A

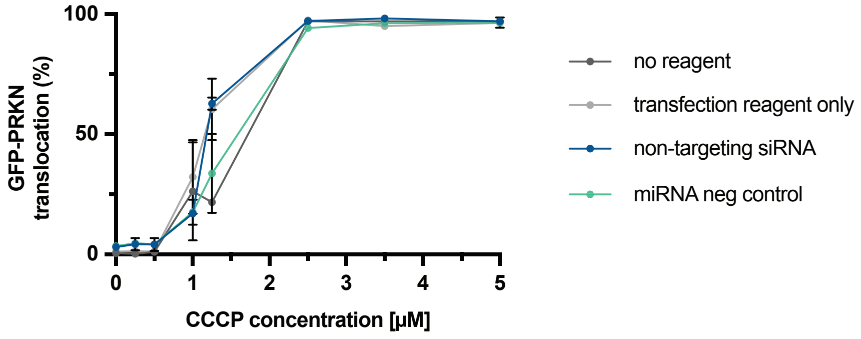
GFP-PRKN
translocation (%)
100
50
0
0
1
2
3
4
5
CCCP concentration [µM]
no reagent
transfection reagent only
non-targeting siRNA
miRNA neg control

B

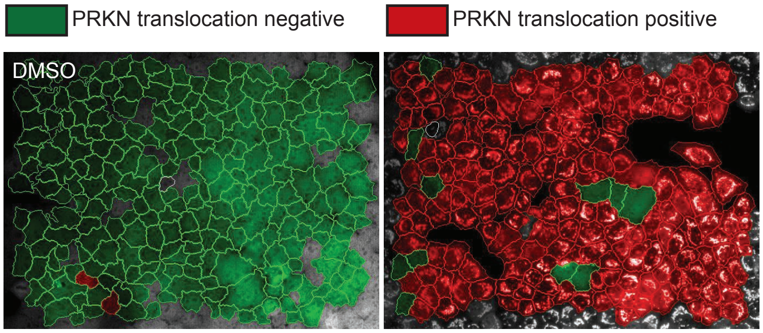
PRKN translocation negative
PRKN translocation positive
DMSO

Figure S2

A

Gene	Fold Change
COL4A1	0.414
HMGCR	0.873
CDC42	0.746
VEGFA	0.976

B

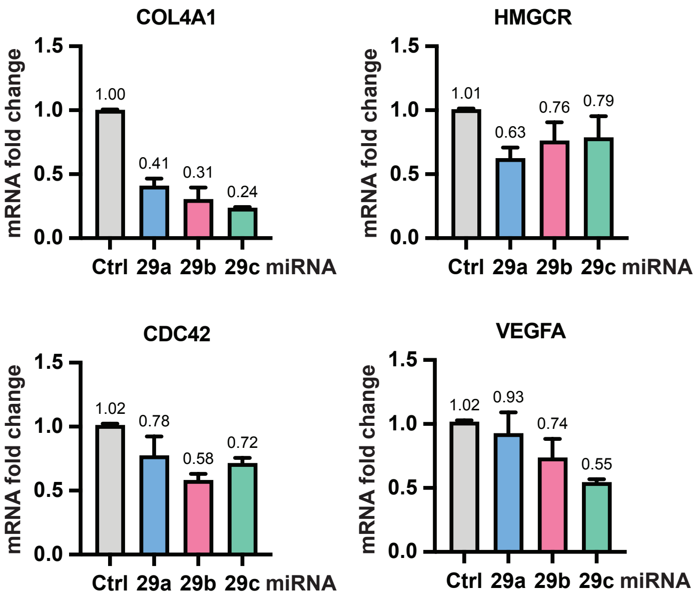

Figure S3

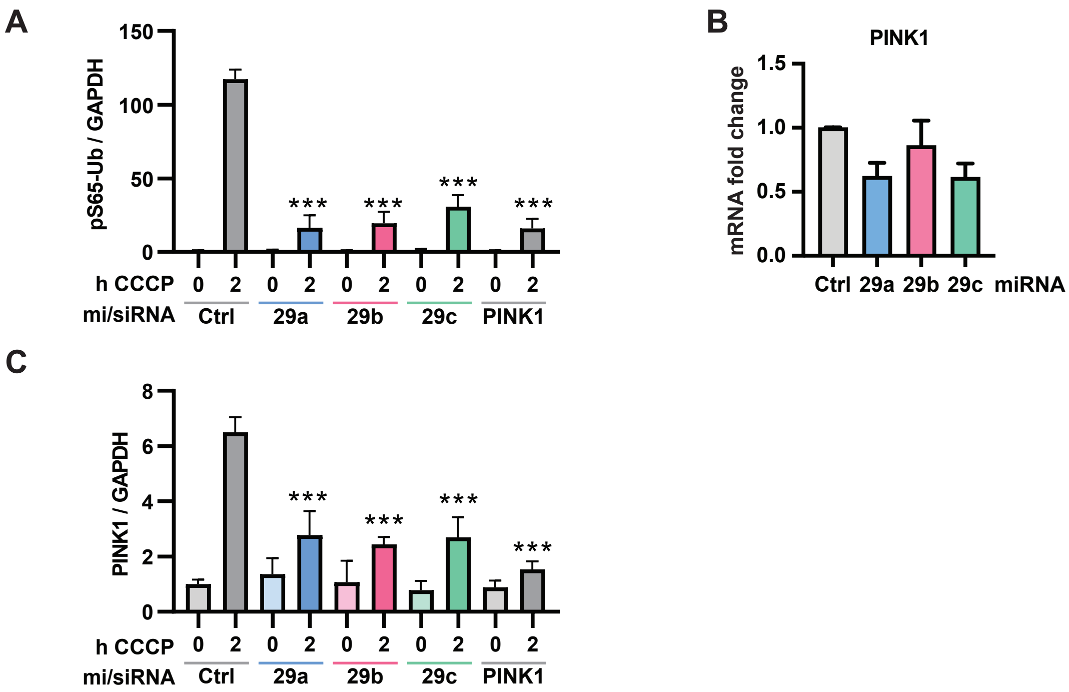
A
pS65-Ub / GAPDH
150
100
50
0
***
***
***
***
h CCCP
0 2 0 2 0 2 0 2 0 2
mi/siRNA
Ctrl
29a
29b
29c
PINK1
B
PINK1
mRNA fold change
1.5
1.0
0.5
0.0
Ctrl 29a 29b 29c miRNA
C
PINK1 / GAPDH
8
6
4
2
0
***
***
***
***
h CCCP
0 2 0 2 0 2 0 2 0 2
mi/siRNA
Ctrl
29a
29b
29c
PINK1

Figure S4

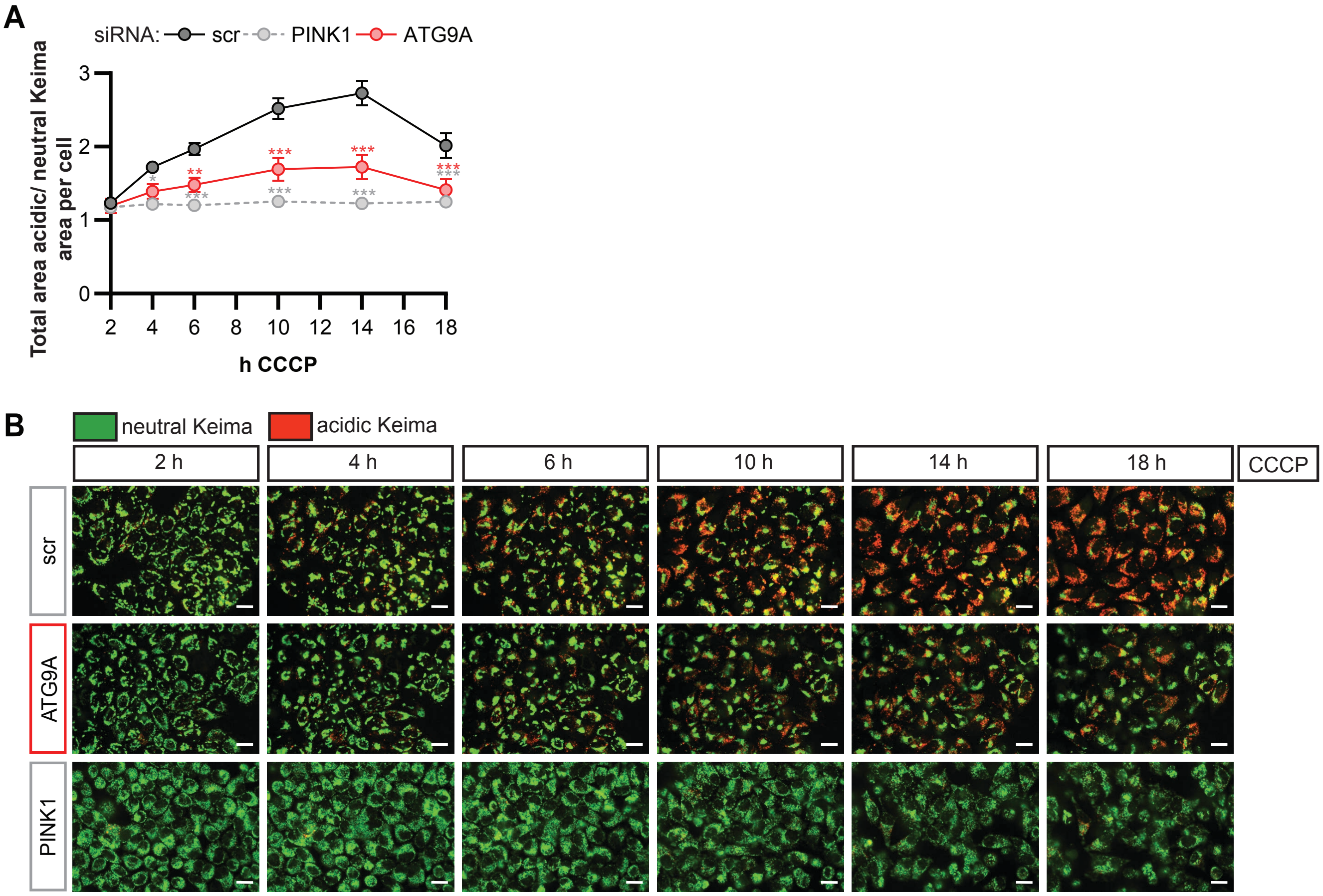

A
siRNA: scr PINK1 ATG9A
Total area acidic/ neutral Keima area per cell
3
2
1
0
2
4
6
8
10
12
14
16
18
h CCCP
B
neutral Keima
acidic Keima
2 h
4 h
6 h
10 h
14 h
18 h
CCCP
scr
ATG9A
PINK1

Figure S5

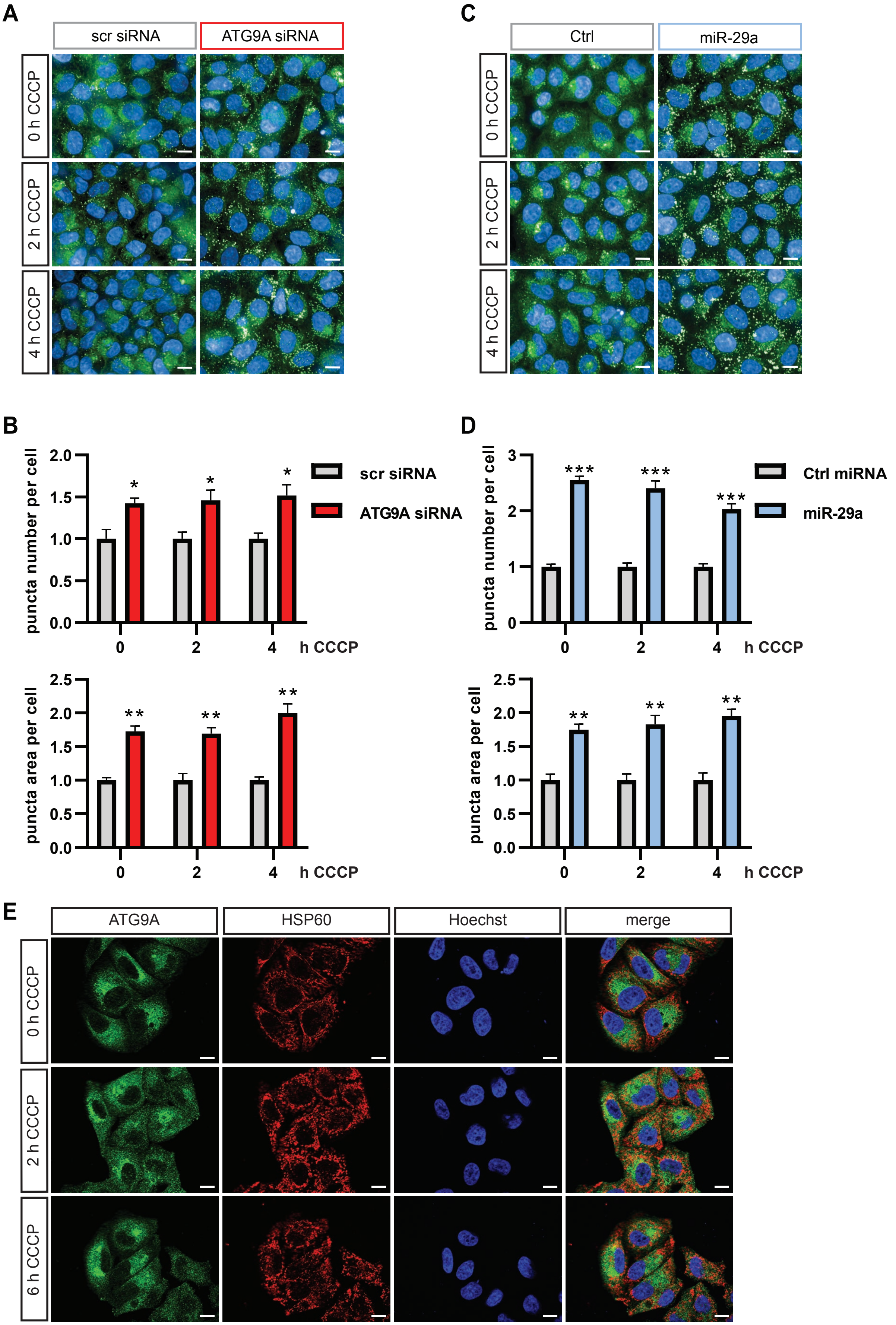

A
scr siRNA
ATG9A siRNA
0 h CCCP
2 h CCCP
4 h CCCP
C
Ctrl
miR-29a
0 h CCCP
2 h CCCP
4 h CCCP
B
puncta number per cell
2.0
1.5
1.0
0.5
0.0
*
*
*
scr siRNA
ATG9A siRNA
0
2
4
h CCCP
puncta area per cell
2.5
2.0
1.5
1.0
0.5
0.0
**
**
**
0
2
4
h CCCP
D
puncta number per cell
3
2
1
0
***
***
***
Ctrl miRNA
miR-29a
0
2
4
h CCCP
puncta area per cell
2.5
2.0
1.5
1.0
0.5
0.0
**
**
**
0
2
4
h CCCP
E
ATG9A
HSP60
Hoechst
merge
0 h CCCP
2 h CCCP
6 h CCCP

Table S1: Imaging properties considered to find the best descriptor for PRKN translocation

415 Properties Considered by PhenoLOGIC's Linear Classifier	
Cell Area [µm²]	a488Cell Radial Mean Ratio SER-Edge
Cell Roundness	a488Cell Profile 1/5 SER-Edge
Cell Ratio Width to Length	a488Cell Profile 2/5 SER-Edge
Nucleus Area [µm²]	a488Cell Profile 3/5 SER-Edge
Intensity Cell Exp2Cam4 Mean	a488Cell Profile 4/5 SER-Edge
Intensity Cell Calculated Image Mean	a488Cell Profile 5/5 SER-Edge
Intensity Cytoplasm Calculated Image Mean	calcCell Symmetry 02
Intensity Cytoplasm Calculated Image StdDev	calcCell Symmetry 03
Intensity Cytoplasm Calculated Image Maximum	calcCell Symmetry 04
Intensity Nucleus Calculated Image Mean	calcCell Symmetry 05
CalcImage Max Cyto/Mean Nuc	calcCell Symmetry 12
Intensity Cell Exp1Cam1 Mean	calcCell Symmetry 13
Intensity Cytoplasm Exp1Cam1 Mean	calcCell Symmetry 14
Intensity Cytoplasm Exp1Cam1 StdDev	calcCell Symmetry 15
Intensity Cytoplasm Exp1Cam1 Maximum	calcCell Threshold Compactness 30%
Intensity Nucleus Exp1Cam1 Mean	calcCell Threshold Compactness 40%
eGFP Max Cyto/Mean Nuc	calcCell Threshold Compactness 50%
a488Cell Symmetry 02	calcCell Threshold Compactness 60%
a488Cell Symmetry 03	calcCell Axial Small Length
a488Cell Symmetry 04	calcCell Axial Length Ratio
a488Cell Symmetry 05	calcCell Radial Mean
a488Cell Symmetry 12	calcCell Radial Relative Deviation
a488Cell Symmetry 13	calcCell Profile 1/5
a488Cell Symmetry 14	calcCell Profile 2/5
a488Cell Symmetry 15	calcCell Profile 3/5
a488Cell Threshold Compactness 30%	calcCell Profile 4/5
a488Cell Threshold Compactness 40%	calcCell Profile 5/5
a488Cell Threshold Compactness 50%	calcCell Symmetry 02 SER-Bright
a488Cell Threshold Compactness 60%	calcCell Symmetry 03 SER-Bright
a488Cell Axial Small Length	calcCell Symmetry 04 SER-Bright
a488Cell Axial Length Ratio	calcCell Symmetry 05 SER-Bright
a488Cell Radial Mean	calcCell Symmetry 12 SER-Bright
a488Cell Radial Relative Deviation	calcCell Symmetry 13 SER-Bright
a488Cell Profile 1/5	calcCell Symmetry 14 SER-Bright
a488Cell Profile 2/5	calcCell Symmetry 15 SER-Bright
a488Cell Profile 3/5	calcCell Threshold Compactness 30% SER-Bright
a488Cell Profile 4/5	calcCell Threshold Compactness 40% SER-Bright
a488Cell Profile 5/5	calcCell Threshold Compactness 50% SER-Bright
a488Cell Symmetry 02 SER-Bright	calcCell Threshold Compactness 60% SER-Bright
a488Cell Symmetry 03 SER-Bright	calcCell Axial Small Length SER-Bright
a488Cell Symmetry 04 SER-Bright	calcCell Axial Length Ratio SER-Bright
a488Cell Symmetry 05 SER-Bright	calcCell Radial Mean SER-Bright
a488Cell Symmetry 12 SER-Bright	calcCell Radial Relative Deviation SER-Bright
a488Cell Symmetry 13 SER-Bright	calcCell Radial Mean Ratio SER-Bright
a488Cell Symmetry 14 SER-Bright	calcCell Profile 1/5 SER-Bright
a488Cell Symmetry 15 SER-Bright	calcCell Profile 2/5 SER-Bright
a488Cell Threshold Compactness 30% SER-Bright	calcCell Profile 3/5 SER-Bright
a488Cell Threshold Compactness 40% SER-Bright	calcCell Profile 4/5 SER-Bright
a488Cell Threshold Compactness 50% SER-Bright	calcCell Profile 5/5 SER-Bright
a488Cell Threshold Compactness 60% SER-Bright	calcCell Symmetry 02 SER-Dark
a488Cell Axial Small Length SER-Bright	calcCell Symmetry 03 SER-Dark
a488Cell Axial Length Ratio SER-Bright	calcCell Symmetry 04 SER-Dark
a488Cell Radial Mean SER-Bright	calcCell Symmetry 05 SER-Dark
a488Cell Radial Relative Deviation SER-Bright	calcCell Symmetry 12 SER-Dark
a488Cell Radial Mean Ratio SER-Bright	calcCell Symmetry 13 SER-Dark
a488Cell Profile 1/5 SER-Bright	calcCell Symmetry 14 SER-Dark
a488Cell Profile 2/5 SER-Bright	calcCell Symmetry 15 SER-Dark
a488Cell Profile 3/5 SER-Bright	calcCell Threshold Compactness 30% SER-Dark
a488Cell Profile 4/5 SER-Bright	calcCell Threshold Compactness 40% SER-Dark
a488Cell Profile 5/5 SER-Bright	calcCell Threshold Compactness 50% SER-Dark
a488Cell Symmetry 02 SER-Dark	calcCell Threshold Compactness 60% SER-Dark
a488Cell Symmetry 03 SER-Dark	calcCell Axial Small Length SER-Dark
a488Cell Symmetry 04 SER-Dark	calcCell Axial Length Ratio SER-Dark
a488Cell Symmetry 05 SER-Dark	calcCell Radial Mean SER-Dark
a488Cell Symmetry 12 SER-Dark	calcCell Radial Relative Deviation SER-Dark
a488Cell Symmetry 13 SER-Dark	calcCell Radial Mean Ratio SER-Dark
a488Cell Symmetry 14 SER-Dark	calcCell Profile 1/5 SER-Dark
a488Cell Symmetry 15 SER-Dark	calcCell Profile 2/5 SER-Dark
a488Cell Threshold Compactness 30% SER-Dark	calcCell Profile 3/5 SER-Dark
a488Cell Threshold Compactness 40% SER-Dark	calcCell Profile 4/5 SER-Dark
a488Cell Threshold Compactness 50% SER-Dark	calcCell Profile 5/5 SER-Dark

a488Cell Threshold Compactness 60% SER-Dark	calcCell Symmetry 02 SER-Ridge
a488Cell Axial Small Length SER-Dark	calcCell Symmetry 03 SER-Ridge
a488Cell Axial Length Ratio SER-Dark	calcCell Symmetry 04 SER-Ridge
a488Cell Radial Mean SER-Dark	calcCell Symmetry 05 SER-Ridge
a488Cell Radial Relative Deviation SER-Dark	calcCell Symmetry 12 SER-Ridge
a488Cell Radial Mean Ratio SER-Dark	calcCell Symmetry 13 SER-Ridge
a488Cell Profile 1/5 SER-Dark	calcCell Symmetry 14 SER-Ridge
a488Cell Profile 2/5 SER-Dark	calcCell Symmetry 15 SER-Ridge
a488Cell Profile 3/5 SER-Dark	calcCell Threshold Compactness 30% SER-Ridge
a488Cell Profile 4/5 SER-Dark	calcCell Threshold Compactness 40% SER-Ridge
a488Cell Profile 5/5 SER-Dark	calcCell Threshold Compactness 50% SER-Ridge
a488Cell Symmetry 02 SER-Ridge	calcCell Threshold Compactness 60% SER-Ridge
a488Cell Symmetry 03 SER-Ridge	calcCell Axial Small Length SER-Ridge
a488Cell Symmetry 04 SER-Ridge	calcCell Axial Length Ratio SER-Ridge
a488Cell Symmetry 05 SER-Ridge	calcCell Radial Mean SER-Ridge
a488Cell Symmetry 12 SER-Ridge	calcCell Radial Relative Deviation SER-Ridge
a488Cell Symmetry 13 SER-Ridge	calcCell Radial Mean Ratio SER-Ridge
a488Cell Symmetry 14 SER-Ridge	calcCell Profile 1/5 SER-Ridge
a488Cell Symmetry 15 SER-Ridge	calcCell Profile 2/5 SER-Ridge
a488Cell Threshold Compactness 30% SER-Ridge	calcCell Profile 3/5 SER-Ridge
a488Cell Threshold Compactness 40% SER-Ridge	calcCell Profile 4/5 SER-Ridge
a488Cell Threshold Compactness 50% SER-Ridge	calcCell Profile 5/5 SER-Ridge
a488Cell Threshold Compactness 60% SER-Ridge	calcCell Symmetry 02 SER-Valley
a488Cell Axial Small Length SER-Ridge	calcCell Symmetry 03 SER-Valley
a488Cell Axial Length Ratio SER-Ridge	calcCell Symmetry 04 SER-Valley
a488Cell Radial Mean SER-Ridge	calcCell Symmetry 05 SER-Valley
a488Cell Radial Relative Deviation SER-Ridge	calcCell Symmetry 12 SER-Valley
a488Cell Radial Mean Ratio SER-Ridge	calcCell Symmetry 13 SER-Valley
a488Cell Profile 1/5 SER-Ridge	calcCell Symmetry 14 SER-Valley
a488Cell Profile 2/5 SER-Ridge	calcCell Symmetry 15 SER-Valley
a488Cell Profile 3/5 SER-Ridge	calcCell Threshold Compactness 30% SER-Valley
a488Cell Profile 4/5 SER-Ridge	calcCell Threshold Compactness 40% SER-Valley
a488Cell Profile 5/5 SER-Ridge	calcCell Threshold Compactness 50% SER-Valley
a488Cell Symmetry 02 SER-Valley	calcCell Threshold Compactness 60% SER-Valley
a488Cell Symmetry 03 SER-Valley	calcCell Axial Small Length SER-Valley
a488Cell Symmetry 04 SER-Valley	calcCell Axial Length Ratio SER-Valley
a488Cell Symmetry 05 SER-Valley	calcCell Radial Mean SER-Valley
a488Cell Symmetry 12 SER-Valley	calcCell Radial Relative Deviation SER-Valley
a488Cell Symmetry 13 SER-Valley	calcCell Radial Mean Ratio SER-Valley
a488Cell Symmetry 14 SER-Valley	calcCell Profile 1/5 SER-Valley
a488Cell Symmetry 15 SER-Valley	calcCell Profile 2/5 SER-Valley
a488Cell Threshold Compactness 30% SER-Valley	calcCell Profile 3/5 SER-Valley
a488Cell Threshold Compactness 40% SER-Valley	calcCell Profile 4/5 SER-Valley
a488Cell Threshold Compactness 50% SER-Valley	calcCell Profile 5/5 SER-Valley
a488Cell Threshold Compactness 60% SER-Valley	calcCell Symmetry 02 SER-Spot
a488Cell Axial Small Length SER-Valley	calcCell Symmetry 03 SER-Spot
a488Cell Axial Length Ratio SER-Valley	calcCell Symmetry 04 SER-Spot
a488Cell Radial Mean SER-Valley	calcCell Symmetry 05 SER-Spot
a488Cell Radial Relative Deviation SER-Valley	calcCell Symmetry 12 SER-Spot
a488Cell Radial Mean Ratio SER-Valley	calcCell Symmetry 13 SER-Spot
a488Cell Profile 1/5 SER-Valley	calcCell Symmetry 14 SER-Spot
a488Cell Profile 2/5 SER-Valley	calcCell Symmetry 15 SER-Spot
a488Cell Profile 3/5 SER-Valley	calcCell Threshold Compactness 30% SER-Spot
a488Cell Profile 4/5 SER-Valley	calcCell Threshold Compactness 40% SER-Spot
a488Cell Profile 5/5 SER-Valley	calcCell Threshold Compactness 50% SER-Spot
a488Cell Symmetry 02 SER-Spot	calcCell Threshold Compactness 60% SER-Spot
a488Cell Symmetry 03 SER-Spot	calcCell Axial Small Length SER-Spot
a488Cell Symmetry 04 SER-Spot	calcCell Axial Length Ratio SER-Spot
a488Cell Symmetry 05 SER-Spot	calcCell Radial Mean SER-Spot
a488Cell Symmetry 12 SER-Spot	calcCell Radial Relative Deviation SER-Spot
a488Cell Symmetry 13 SER-Spot	calcCell Radial Mean Ratio SER-Spot
a488Cell Symmetry 14 SER-Spot	calcCell Profile 1/5 SER-Spot
a488Cell Symmetry 15 SER-Spot	calcCell Profile 2/5 SER-Spot
a488Cell Threshold Compactness 30% SER-Spot	calcCell Profile 3/5 SER-Spot
a488Cell Threshold Compactness 40% SER-Spot	calcCell Profile 4/5 SER-Spot
a488Cell Threshold Compactness 50% SER-Spot	calcCell Profile 5/5 SER-Spot
a488Cell Threshold Compactness 60% SER-Spot	calcCell Symmetry 02 SER-Hole
a488Cell Axial Small Length SER-Spot	calcCell Symmetry 03 SER-Hole
a488Cell Axial Length Ratio SER-Spot	calcCell Symmetry 04 SER-Hole
a488Cell Radial Mean SER-Spot	calcCell Symmetry 05 SER-Hole
a488Cell Radial Relative Deviation SER-Spot	calcCell Symmetry 12 SER-Hole
a488Cell Radial Mean Ratio SER-Spot	calcCell Symmetry 13 SER-Hole
a488Cell Profile 1/5 SER-Spot	calcCell Symmetry 14 SER-Hole
a488Cell Profile 2/5 SER-Spot	calcCell Symmetry 15 SER-Hole

a488Cell Profile 3/5 SER-Spot	calcCell Threshold Compactness 30% SER-Hole
a488Cell Profile 4/5 SER-Spot	calcCell Threshold Compactness 40% SER-Hole
a488Cell Profile 5/5 SER-Spot	calcCell Threshold Compactness 50% SER-Hole
a488Cell Symmetry 02 SER-Hole	calcCell Threshold Compactness 60% SER-Hole
a488Cell Symmetry 03 SER-Hole	calcCell Axial Small Length SER-Hole
a488Cell Symmetry 04 SER-Hole	calcCell Axial Length Ratio SER-Hole
a488Cell Symmetry 05 SER-Hole	calcCell Radial Mean SER-Hole
a488Cell Symmetry 12 SER-Hole	calcCell Radial Relative Deviation SER-Hole
a488Cell Symmetry 13 SER-Hole	calcCell Radial Mean Ratio SER-Hole
a488Cell Symmetry 14 SER-Hole	calcCell Profile 1/5 SER-Hole
a488Cell Symmetry 15 SER-Hole	calcCell Profile 2/5 SER-Hole
a488Cell Threshold Compactness 30% SER-Hole	calcCell Profile 3/5 SER-Hole
a488Cell Threshold Compactness 40% SER-Hole	calcCell Profile 4/5 SER-Hole
a488Cell Threshold Compactness 50% SER-Hole	calcCell Profile 5/5 SER-Hole
a488Cell Threshold Compactness 60% SER-Hole	calcCell Symmetry 02 SER-Saddle
a488Cell Axial Small Length SER-Hole	calcCell Symmetry 03 SER-Saddle
a488Cell Axial Length Ratio SER-Hole	calcCell Symmetry 04 SER-Saddle
a488Cell Radial Mean SER-Hole	calcCell Symmetry 05 SER-Saddle
a488Cell Radial Relative Deviation SER-Hole	calcCell Symmetry 12 SER-Saddle
a488Cell Radial Mean Ratio SER-Hole	calcCell Symmetry 13 SER-Saddle
a488Cell Profile 1/5 SER-Hole	calcCell Symmetry 14 SER-Saddle
a488Cell Profile 2/5 SER-Hole	calcCell Symmetry 15 SER-Saddle
a488Cell Profile 3/5 SER-Hole	calcCell Threshold Compactness 30% SER-Saddle
a488Cell Profile 4/5 SER-Hole	calcCell Threshold Compactness 40% SER-Saddle
a488Cell Profile 5/5 SER-Hole	calcCell Threshold Compactness 50% SER-Saddle
a488Cell Symmetry 02 SER-Saddle	calcCell Threshold Compactness 60% SER-Saddle
a488Cell Symmetry 03 SER-Saddle	calcCell Axial Small Length SER-Saddle
a488Cell Symmetry 04 SER-Saddle	calcCell Axial Length Ratio SER-Saddle
a488Cell Symmetry 05 SER-Saddle	calcCell Radial Mean SER-Saddle
a488Cell Symmetry 12 SER-Saddle	calcCell Radial Relative Deviation SER-Saddle
a488Cell Symmetry 13 SER-Saddle	calcCell Radial Mean Ratio SER-Saddle
a488Cell Symmetry 14 SER-Saddle	calcCell Profile 1/5 SER-Saddle
a488Cell Symmetry 15 SER-Saddle	calcCell Profile 2/5 SER-Saddle
a488Cell Threshold Compactness 30% SER-Saddle	calcCell Profile 3/5 SER-Saddle
a488Cell Threshold Compactness 40% SER-Saddle	calcCell Profile 4/5 SER-Saddle
a488Cell Threshold Compactness 50% SER-Saddle	calcCell Profile 5/5 SER-Saddle
a488Cell Threshold Compactness 60% SER-Saddle	calcCell Symmetry 02 SER-Edge
a488Cell Axial Small Length SER-Saddle	calcCell Symmetry 03 SER-Edge
a488Cell Axial Length Ratio SER-Saddle	calcCell Symmetry 04 SER-Edge
a488Cell Radial Mean SER-Saddle	calcCell Symmetry 05 SER-Edge
a488Cell Radial Relative Deviation SER-Saddle	calcCell Symmetry 12 SER-Edge
a488Cell Radial Mean Ratio SER-Saddle	calcCell Symmetry 13 SER-Edge
a488Cell Profile 1/5 SER-Saddle	calcCell Symmetry 14 SER-Edge
a488Cell Profile 2/5 SER-Saddle	calcCell Symmetry 15 SER-Edge
a488Cell Profile 3/5 SER-Saddle	calcCell Threshold Compactness 30% SER-Edge
a488Cell Profile 4/5 SER-Saddle	calcCell Threshold Compactness 40% SER-Edge
a488Cell Profile 5/5 SER-Saddle	calcCell Threshold Compactness 50% SER-Edge
a488Cell Symmetry 02 SER-Edge	calcCell Threshold Compactness 60% SER-Edge
a488Cell Symmetry 03 SER-Edge	calcCell Axial Small Length SER-Edge
a488Cell Symmetry 04 SER-Edge	calcCell Axial Length Ratio SER-Edge
a488Cell Symmetry 05 SER-Edge	calcCell Radial Mean SER-Edge
a488Cell Symmetry 12 SER-Edge	calcCell Radial Relative Deviation SER-Edge
a488Cell Symmetry 13 SER-Edge	calcCell Radial Mean Ratio SER-Edge
a488Cell Symmetry 14 SER-Edge	calcCell Profile 1/5 SER-Edge
a488Cell Symmetry 15 SER-Edge	calcCell Profile 2/5 SER-Edge
a488Cell Threshold Compactness 30% SER-Edge	calcCell Profile 3/5 SER-Edge
a488Cell Threshold Compactness 40% SER-Edge	calcCell Profile 4/5 SER-Edge
a488Cell Threshold Compactness 50% SER-Edge	calcCell Profile 5/5 SER-Edge
a488Cell Threshold Compactness 60% SER-Edge	eGFP Cytoplasm Threshold Comp Threshold Compactness 30%
a488Cell Axial Small Length SER-Edge	eGFP Cytoplasm Threshold Comp Threshold Compactness 40%
a488Cell Axial Length Ratio SER-Edge	eGFP Cytoplasm Threshold Comp Threshold Compactness 50%
a488Cell Radial Mean SER-Edge	eGFP Cytoplasm Threshold Comp Threshold Compactness 60%
a488Cell Radial Relative Deviation SER-Edge	

Table S2: Image analysis routine for miRNA screen

Building Block	Purpose
1. Find Image Region	Identifies the Whole Image as a Population and Region.
2. Calculate Morphology	Calculates the Area [µm2] of the Whole Image
3. Find Image Region	Finds Regions covered by Green (Exp1Cam1)
4. Calculate Morphology	Calculates the Area [µm2] of the GreenArea
5. Calculate Image	Normalized Green Channel minus Normalized Blue Channel, yields Green signal
6. Find Nuclei	Finds Nuclei. Initial Population: DAPI_Pos
7. Find Cytoplasm	Add Cell Regions to DAPI_Pos
8. Select Population	Remove Cells that touch the image border. Output Population: Nuclei_Border_Filtered
9. Calculate Morphology	Calculates the Area [µm2], Roundness and Length-to-Width Ratio of the Nuclei_Border_Filtered, Cell
10. Calc. Morphology Properties	Calculates the Area [µm2] of the Nuclei_Border_Filtered, Nucleus Region
11. Select Population	Selects Nuclei_Border_Filtered Objects based on Cell Roundness, Cell Area [µm2], and Nucleus Area
12. Calculate Intensity Properties	Calculates Blue Intensity (Exp2Cam4) in the Cell Region of Nuclei
13. Calculate Intensity Properties	Calculates ‘Calculated Image’ Intensity in Cell Region of Nuclei
14. Calculate Intensity Properties	Calculates ‘Calculated Image’ Intensity in Cytoplasm Region of Nuclei
15. Calculate Intensity Properties	Calculates ‘Calculated Image’ Intensity in Nucleus Region of Nuclei
16. Calculate Properties	(Intensity Cytoplasm Calculated Image Max.) / (Intensity Nucleus Calculated Image Mean)
17. Calculate Intensity Properties	Calculates Green Intensity (Exp1Cam1) in the Cell Region of Nuclei
18. Calculate Intensity Properties	Calculates Green Intensity (Exp1Cam1) in the Cytoplasm Region of Nuclei
19. Calculate Intensity Properties	Calculates Green Intensity (Exp1Cam1) in the Nucleus Region of Nuclei
20. Calculate Properties	(Intensity Cytoplasm Exp1Cam1 Max.) / (Intensity Nucleus Exp1Cam1 Mean)
21. Calculate Morphology	STAR Morphology with Texture Features, Exp1Cam1 Channel, Cell Region of Nuclei
22. Calculate Morphology	STAR Morphology with Texture Features, ‘Calculated Image’ Channel, Cell Region of Nuclei
23. Calculate Morphology	Threshold Compactness, Exp1Cam1(Green) of Cytoplasm Region of Nuclei
24. Select Population	Linear Classifier on Nuclei, 415 properties considered
	Output Population A : DMSO
	Output Population B : 3.5_CCCP
	Output Population C : InBetween
25. Calculate Properties	By Related Populations
	Relates the Area [µm2] of the Population pGreenAreas to the Population Whole Image
26. Calculate Properties	By Related Populations
	Relates the Cell Area [µm2] of the Population Nuclei to the Population Whole Image
27. Calculate Properties	Percentage of the Whole Image Area [µm2] covered by pGreenAreas.
	((rGreenArea Area-Sum per Object) / (Whole Image Region Area))*100
28. Calculate Properties	Percentage of the Whole Image Area [µm2] covered by segmented Cells.
	((Cell Area-Sum per Object) / (Whole Image Region Area))*100
29. Select Population	Flags fields of view less than 50% confluent.
30. Define Results	Specifies data to be included in the final output table.

Table S3: Final linear classifier to define PRKN translocation positive cells

Goodness: 5.21

Offset: -7.42822

Properties (ordered by relevance)	Linear Coefficient
a488Cell Threshold Compactness 40%	-22.3388
a488Cell Profile 4/5 SER-Dark	-135.71
a488Cell Profile 3/5 SER-Bright	91.583
eGFP Cytoplasm Threshold Comp Threshold Compactness 40%	22.9283
a488Cell Threshold Compactness 50%	-14.4039
a488Cell Profile 3/5 SER-Edge	32.0958
calcCell Profile 3/5	2.23318
calcCell Threshold Compactness 60% SER-Edge	9.08955
a488Cell Profile 3/5 SER-Saddle	75.3239
eGFP Max Cyto/Mean Nuc	0.945125
calcCell Radial Mean SER-Ridge	-0.167702
a488Cell Radial Mean Ratio SER-Valley	-5.2088

Table S4: conserved 3'-UTR miR-29 binding sites and alignment of ATG9A to miR-29a/b/c.

miRNA	3'-UTR Binding Site	Predicted Alignment of ATG9A (top) to miR-29 (bottom)	Cumulative weighted context++ score	Aggregate P_{CT}
**miR-29a**	251-258	5' ...CCUUGGCUCAGAGUGUGGUGCUA...<br>||||||| 3' AUUGGCUAAAGUCUACCACGAU	-0.28	0.88
**miR-29b**	251-258	5' ...CCUUGGCUCAGAGUGUGGUGCUA...<br>||||||| 3' UUGUGACUAAAGUUUACCACGAU	-0.26	0.88
**miR-29c**	251-258	5' ...CCUUGGCUCAGAGUGUGGUGCUA...<br>||||||| 3' AUUGGCUAAAGUUUACCACGAU	-0.26	0.88

Data obtained from TargetScan version 7.1.

Table S5: ATG9A species alignment at the R631 and S828 positions

Species	R631	S828
Homo sapiens	AGSSCRGPPLP	VPEEGSEDELP
Pongo abelii	AGSSCRGPPLP	VPEEGSEDELP
Mus musculus	AGSSCRGPSLS	VPEEGSEDELP
Sus scrofa	AGSSCRGPPLP	VPEEGSEDELP
Rattus norvegicus	AGSSCRGPPLS	VPEEGSEDELP
Bos taurus	AGSSCRGPPLP	VPEEGSEDELP

Data obtained from UniProt. R631 and S828 are highlighted in red.
